# A 4D view on the evolution of metamorphic dehydration reactions

**DOI:** 10.1038/s41598-017-07160-5

**Published:** 2017-07-31

**Authors:** John Bedford, Florian Fusseis, Henri Leclère, John Wheeler, Daniel Faulkner

**Affiliations:** 1 Department of Earth, Ocean and Ecological Sciences, University of Liverpool, Liverpool, L69 3GP UK; 2 School of Geosciences, University of Edinburgh, Edinburgh, EH9 3JW UK

## Abstract

Metamorphic reactions influence the evolution of the Earth’s crust in a range of tectonic settings. For example hydrous mineral dehydration in a subducting slab can produce fluid overpressures which may trigger seismicity. During reaction the mechanisms of chemical transport, including water expulsion, will dictate the rate of transformation and hence the evolution of physical properties such as fluid pressure. Despite the importance of such processes, direct observation of mineral changes due to chemical transport during metamorphism has been previously impossible both in nature and in experiment. Using time-resolved (4D) synchrotron X-ray microtomography we have imaged a complete metamorphic reaction and show how chemical transport evolves during reaction. We analyse the dehydration of gypsum to form bassanite and H_2_O which, like most dehydration reactions, produces a solid volume reduction leading to the formation of pore space. This porosity surrounds new bassanite grains producing fluid-filled moats, across which transport of dissolved ions to the growing grains occurs via diffusion. As moats grow in width, diffusion and hence reaction rate slow down. Our results demonstrate how, with new insights into the chemical transport mechanisms, we can move towards a more fundamental understanding of the hydraulic and chemical evolution of natural dehydrating systems.

## Introduction

The study of metamorphism is underpinned by thermodynamics; that a system will tend towards a state of minimum energy and reach equilibrium with its environment^1, 2^. However there is widespread evidence of disequilibrium textures on the sub-millimeter scale in crustal rocks including mineral zoning, coexisting polymorphs and reaction rims^3–5^. This shows that kinetic impediments often prevent thermodynamic equilibrium from being reached completely. Knowledge of kinetic processes, such as chemical transport and attachment/detachment of atoms at mineral interfaces, is vital to understand the controls on reaction rate and is also important for characterizing mid-crustal fluid flow as transformations often involve the release of fluids (e.g. dehydration reactions). Dehydration reactions are abundant in the crust during prograde metamorphism, particularly in subduction zone settings where they are thought to play an important role in generating intermediate-depth seismicity^6–11^ and also in returning stored water from the oceanic lithosphere back to the surface^12, 13^. If fluids are unable to drain, the resulting high fluid pressures have been previously shown to slow the reaction rate^14^, thus the pressure evolution is coupled to the reaction kinetics. The kinetic controls on reaction are dependent upon the pathways^15^ that chemical components take from detachment at the reactant phase to incorporation into the lattice of the product mineral(s). It is impossible to observe directly these pathways in action in rocks or in traditional experimental setups; therefore our understanding of reaction pathways is limited to disequilibrium textures preserved after reaction^16^.

The use of 4D synchrotron X-ray microtomography provides new opportunities in the experimental investigation of metamorphism by allowing direct microstructural and mineralogical information to be gathered on the micron scale as a reaction proceeds. We conducted a confined heating experiment to investigate the dehydration of gypsum (CaSO_4_•2H_2_O) to form bassanite (CaSO_4_•0.5H_2_O) and H_2_O. The reaction was documented in a 3-dimensional X-ray microtomographic time series dataset (ie. 4D data) using an X-ray transparent hydrothermal cell^17^ that was installed in the microtomography beamline 2BM at the Advanced Photon Source (USA). Gypsum dehydration has proven to be analogous to reactions involving silicate minerals^18^ but is much more suitable for synchrotron study since it completes in hours rather than years and begins at 100 °C, which is much lower than most silicate dehydration reactions^19^. The univariant gypsum to bassanite transition is also relatively simple when compared to many silicate dehydration reactions which often involve solid solution series. The fine-grained starting material from Volterra, Italy, has been widely used in studies of dehydration as it is relatively homogeneous with initially low porosities of <1%^11, 18, 20–22^. Like most dehydration reactions, the breakdown of gypsum involves a reduction in the solid molar volume, which leads to the formation of porosity (29% when fully dehydrated). However the reaction is associated with a net volume increase as the water produced has a greater volume than the pore space. This means that some water must be expelled, but if that is not possible then the fluid pressure will increase, slowing down the reaction^14^. Thus there is a feedback between the mechanisms of reaction and fluid expulsion.

A cylindrical sample (2 mm diameter, 5 mm length) was subject to 9 MPa confining pressure (Pc), 4 MPa pore fluid pressure (Pf) and held at 115 °C for approximately 9 hours. The difference between the two pressures (Pc − Pf = 5 MPa), is small, analogous to the situation in natural dehydrating systems^23^. There are no macroscopic differential stresses in this setup; however it is worth noting that anisotropic grain-scale stresses do arise when the confining pressure and fluid pressure are not equal^24^. Importantly, at the low effective pressure in our experiment there is no pore collapse by compaction and the evolving microstructure is produced by reaction alone. The experiment therefore represents an end member condition; in natural settings compaction may occur if excess fluid is able to drain leading to an increase in effective pressure acting on the reacting rocks. During the experiment, 3-dimensional microtomographic datasets of the entire sample were acquired in 15 minute intervals (see methods). The X-ray microtomographic data have a voxel size of 1.3 μm which is sufficient to image growing grains in detail, and the contrast in absorption allows for segmentation (automatic recognition) of the evolving pore space as it is distinct from the solid phases.

## Results and Discussion

**Figure 1:**
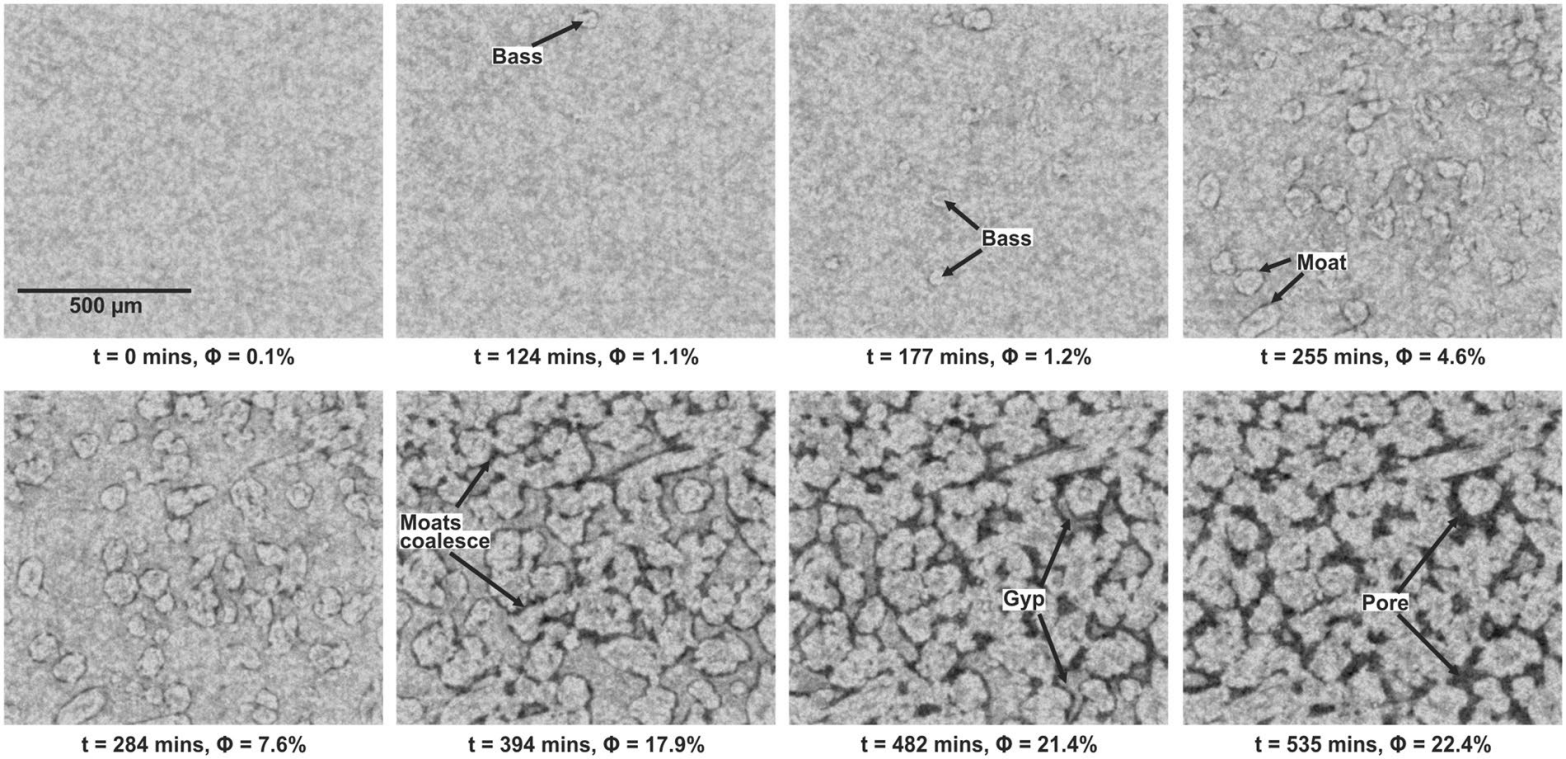
Time-series microtomographic reconstructions of the dehydrating gypsum sample. The first bassanite grains are observed after 124 minutes. The new grains are evenly distributed throughout the sample and grow surrounded by moats of porosity which appear black in the reconstructions. The grains grow larger and the porosity (Φ) increases as the moats get wider. At 394 minutes the grains begin to impinge on each other and the porous moats start to coalesce. By 482 minutes the bassanite grains isolate the remaining pockets of gypsum which is consumed in the reaction and the pores expand into this space.

Figure 1 shows a selection of time sequence micrographs highlighting the evolution of the gypsum and the growing bassanite grains in conjunction with the evolving porosity in the sample (see also Supplementary Movie 1). The first, relatively isolated, grains of bassanite that we observe appear after approximately 120 minutes, each surrounded by newly formed pore space. Pores initially wrap around bassanite grains forming a fluid-filled, moat-like structure (Fig. 1). As the reaction continues the grains and pores grow larger until grains begin to impinge on each other and the moats coalesce.

**Figure 2:**
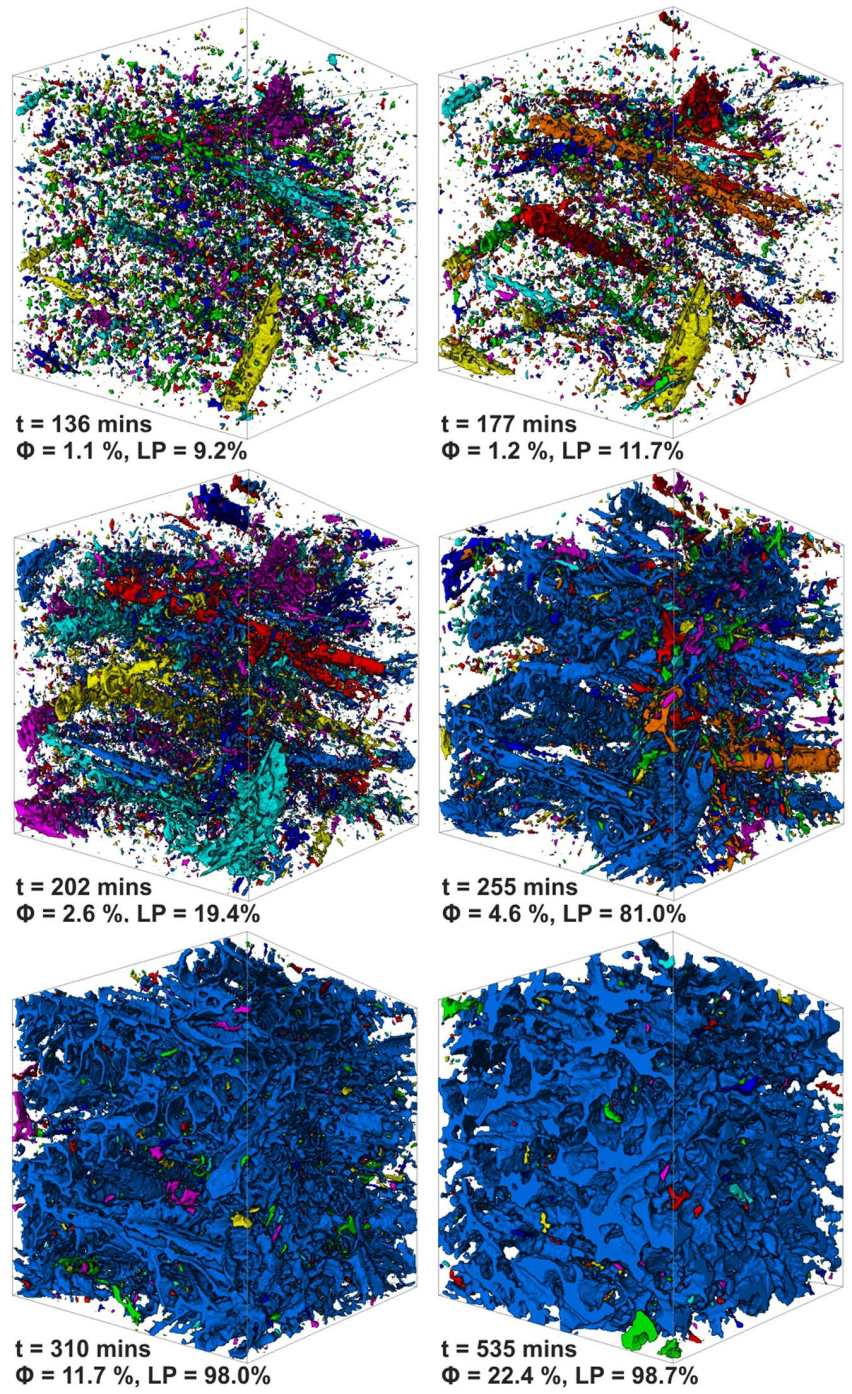
3-dimensional reconstructions of the pore network with time. Different pore clusters appear in different colours in the reconstructions. The analysed subvolume is a cube with a side length of 975 μm. Until 177 minutes there are thousands of isolated pores, with the largest pore cluster (LP) only comprising 11.7% of the pore network at this time. By 202 minutes the largest pores are beginning to expand and by 255 minutes most of them are interconnected with the largest pore cluster (which appears blue) comprising 81% of the total pore network. After 310 minutes this pore cluster dominates the drainage architecture (98%) with only a few isolated small pores remaining.

During the experiment the total porosity in the sample increases non-linearly until it begins to settle at just below 25% after about 450 minutes (see Supplementary Fig. 1). Interconnection of individual moats rapidly leads to the formation of a complex pore cluster between 202 and 255 minutes into the experiment. This cluster percolates throughout the entire analyzed subvolume (Fig. 2, Supplementary Movie 2). Our analysis of the pore size distribution in the sample reveals that, up to 202 minutes, the largest pore cluster accounts for about 20% of the total porosity. By 255 minutes this value has risen to approximately 81% (Fig. 2, Supplementary Fig. 2), showing a dramatic increase in connectivity. The overall porosity in the sample at this time is only 4.6%, indicating that efficient expulsion of H_2_O is achievable after a relatively small amount of reaction. This stage coincides with a consolidation of the pore structure, where small isolated pores are progressively incorporated into a sample-scale drainage architecture (Fig. 2, Supplementary Fig. 3). A recent study, modelling fluid expulsion during serpentinite dehydration, has shown that early connectivity of the reaction generated porosity is key for initiating fluid channelization in a subducting slab^13^, which is thought to be an important mechanism in allowing large-scale fluid expulsion. Our results are in agreement with this finding and therefore have wider implications for the hydraulics of a subduction zone, suggesting that efficient fluid expulsion may be achieved in the initial stages of a dehydration reaction.

**Figure 3:**
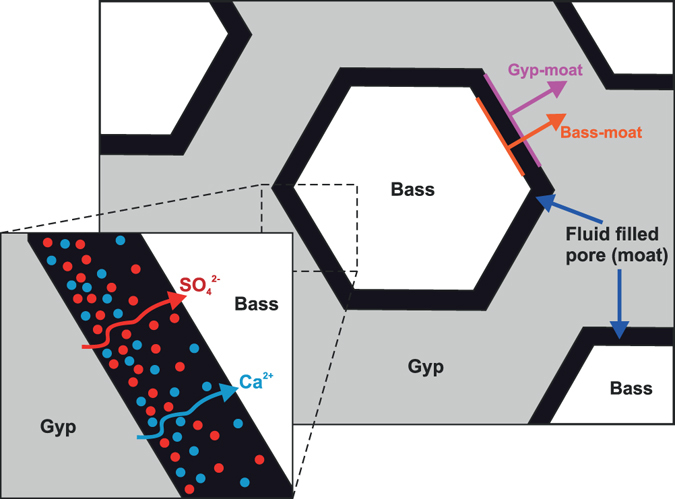
Schematic cartoon of the chemical transport pathways during reaction. For the majority of their growth history bassanite grains grow in relative isolation from nearby grains. Transport of dissolved solutes (Ca^2+^ and SO_4_^2−^) occurs via diffusion across the fluid-filled moats surrounding the growing bassanite grains. Also marked are the two interfaces that were measured in the 2D image analysis (Fig. 4): the bassanite-moat interface and the gypsum-moat interface. As grains grow both interfaces move away from the grain centre and we are able to track how the area inside these interfaces evolves with time.

**Figure 4:**
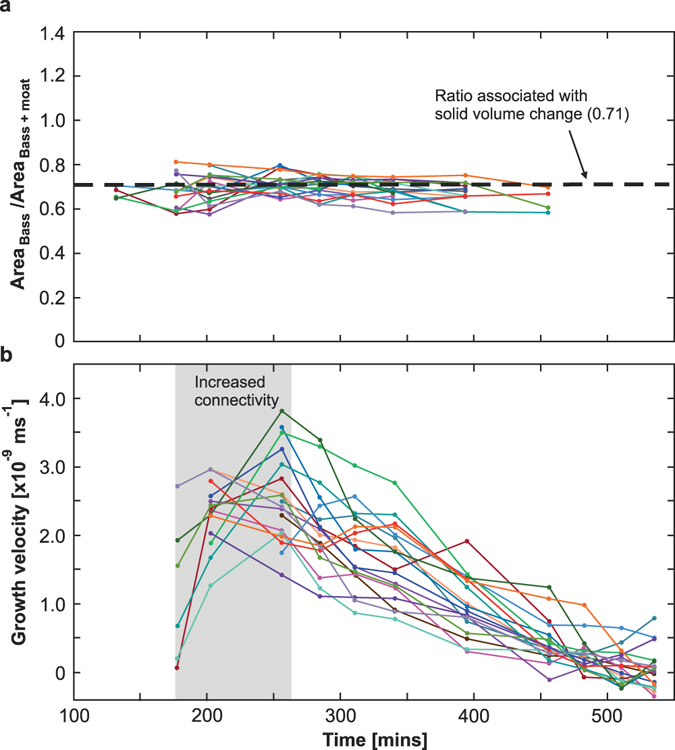
Quantification of the grain areas and growth velocities with time. (**a**) Ratio of the areas between the grain and the grain + moat. Different curves represent individual grains and their associated moats. Values oscillate around a central ratio of 0.71 which is equivalent to the solid volume change associated with the reaction (i.e. molar volume of bassanite/molar volume of gypsum = 0.71). Once the moats begin to coalesce they can no longer be associated to a given grain and therefore the ratio can no longer be analysed, hence why the curves do not continue for the duration of the experiment. (**b**) Grain growth velocity curves for individual bassanite grains. The grey shaded area represents the time when the connectivity of the sample dramatically increases. This corresponds to an initial acceleration in growth velocity for the majority of grains. Once the drainage architecture has established itself the growth velocities decrease with time, which is associated with diffusion of solutes across the moats (Fig. 3).

In metamorphism, the rate limiting processes are typically considered to be diffusion^5, 25, 26^, mineral interface reactions^27–29^ or combinations of both depending on the length scale^30^. In the case of the gypsum to bassanite transformation, in order for grain growth to occur, bassanite grains require a supply of Ca^2+^ and SO_4_^2−^ ions. It is often assumed that the transport of dissolved chemical components during metamorphism occurs through a thin grain-boundary fluid film^25^. However solid volume changes during a transformation produce transient porosity and our datasets show that the transport of ions must occur via diffusion through the wide moats produced by the reaction itself (Fig. 3). As the bassanite grains are elongate, we expect the areal growth rate to approximate to the volumetric growth rate. We performed 2D image analysis to measure the areas (A) of the bassanite grains and the grains + moats. Figure 4a shows that the ratio of these areas clusters around a central value of approximately 0.71 before the moats coalesce and can no longer be attributed to individual grains. This ratio of 0.71 corresponds exactly to the solid volume change associated with the reaction, indicating that each grain-moat pair is evolving as a closed chemical system with respect to Ca^2+^ and SO_4_^2−^, in relative isolation from nearby grains. It also suggests that advection of the dissolved solutes is negligible even though the excess H_2_O is able to dissipate away from the site of reaction. The reaction pathway must therefore involve dissolution at the gypsum-moat interface, diffusion across the moat and precipitation at the bassanite-moat interface (Fig. 3). Previous experimental work^31^ and observations on natural samples^13^ have shown similar moat like structures around forsterite grains in partially dehydrated serpentinite, suggesting this reaction pathway may be dominant in many dehydration reactions.

We also derived approximate growth velocities (u) for individual grains from $u=\,\frac{1}{p}\frac{dA}{dt}$ where *t* is time, p is the perimeter and A is the area of a given grain. Most grains show an initial increase in velocity until about 250 minutes before an overall deceleration is observed for the majority of their growth history (Fig. 4b). The initial growth acceleration is associated with the increasing connectivity of the sample. Prior to the pore network becoming interconnected, locally high pore-fluid pressure in isolated pores hinders the reaction. Previous experimental work on gypsum dehydration has shown that reaction rate is highly dependent on the pore fluid pressure^14^. There is a strong agreement between this previous dataset and the reaction rate observed under the experimental conditions of this study (see Supplementary equations and Supplementary Figure 4), highlighting the importance of pore fluid pressure as a rate–controlling parameter. The acceleration in grain growth coincides with the rapid increase in connectivity between 202–255 minutes (Fig. 2) because the excess pore fluid pressure, which slows the reaction, is able to dissipate. The subsequent deceleration in growth through time is what would be expected for a diffusional control on reaction, as recently documented for a reaction involving fluid-solid interactions but not dehydration^32^. As our moat widths increase, the diffusion distance lengthens causing the growth rates to slow. We calculate a bulk diffusion coefficient of 1.23 × 10^−10^ m^2^/s for the transport of chemical components across the moats (see Supplementary equations and Supplementary Figure 5).

On a practical level, these findings show that the solid volume changes that occur as a result of dehydration can create the main pathways to facilitate the mass transport of both hydrous and dissolved chemical components during reaction, albeit on different scales. The new porosity provides a route for excess H_2_O to escape in the early stages of the reaction and also generates diffusion gradients along which the dissolved solutes migrate to the growing grains. Identification of the main transport pathway has important implications for the understanding of how reactions will interact with other processes such as deformation, which will no doubt reduce the available pore space and in turn restrict the ability for fluids to be expelled while also reducing the diffusion distances that govern the reaction rate. Knowledge of the kinetic controls on reaction is paramount, particularly for the modelling of dehydrating systems, as they will determine the overall rate of transformation and in turn have implications for the mechanical and hydraulic evolution of the system. We have shown that the reaction rate can be controlled by both the fluid pressure and the diffusion of dissolved solutes, and that this is determined by the hydraulic properties of the dehydrating rock. Finally, our results can help identify scenarios when seismicity might occur in subduction zones. In our case, if the transient porosity is maintained during reaction, then the early expulsion of fluids and slowing reaction rate suggest that greatest chance of seismicity is early in the reaction rather than at it maximum rate.

## Methods

### Sample preparation

The gypsum samples were cored from a precision ground slab of alabaster. The slab originated from a block of polycrystalline Volterra gypsum which has grain sizes in the range of 10–200 μm^11^. Volterra gypsum is considered to be fairly isotropic; however weak shape preferred orientations have been reported^33^. The slab was cut into a 6 cm × 6 cm square and ground to a thickness of 5 mm to a tolerance of 100 μm. Cores, 2 mm in diameter, were drilled from the slab and the ends were lightly hand-ground to remove any roughness produced during coring.

### Microtomographic data acquisition

Experiments were conducted at beam line 2BM at the Advanced Photon Source (APS), in the upstream hutch 25 m from the source. There, a polychromatic beam filtered by 35 mm borosilicate glass yielded a photon flux with an energy peak at 65 KeV. A Cooke pco.edge sCMOS camera with 2560 × 2160 pixels (pixel size 6.5 × 6.5 μm^2^) was used in a flying scan mode, where projections are recorded while the sample is continuously rotated (i.e. the stage rotation does not stop in between image acquisitions). The sample-detector distance was kept to 80 mm to minimise phase contrast in the data. For the experiment we used an x-ray transparent Hassler core holder^17^, where the sample was pressurised and heated to 388 K after an initial reference scan. The camera recorded projections from a 10 μm thick LuAG:Ce single crystal scintillator, magnified through a 5x Mitutoyo long-working distance lens yielding a pixel size of 1.3 µm. Projections were collected with an exposure time of 50 ms while the sample was rotated over 180° with 1.2 °/s. 1500 projections were collected in 150 s. Each time step comprises three individual scans acquired back to back at three different vertical positions to cover the entire sample cylinder. The entire sample was scanned every 15 minutes over a total of 9 hours.

### Data processing and analysis

Microtomographic data were reconstructed from the projections using TomoPy^34^. The reconstructed data were processed using the commercial software package Avizo 8.0 and the open source software Fiji^35^. The reconstructed image stacks were cropped (750 × 750 × 750 voxels) and processed to reduce image noise using a non-local means filter. Subsequently, the porosity was segmented from the data in Fiji using the Trainable Weka Segmentation Algorithm (http://imagej.net/Trainable_Weka_Segmentation). The segmented porosity was labelled and analyzed in Avizo to allow for a detailed estimation of porosity and percolation in the time series data. Porosity measurements are based on the segmented datasets and reflect the systematic evolution of the porosity in the sample during reaction.

## Electronic supplementary material

Supplementary Information

Supplementary movie 1

Supplementary movie 2
