# Supplementary material for: A 4D view on the evolution of metamorphic dehydration reactions
Source: Sci Rep. 2017 Jul 31;7:6881. doi: 10.1038/s41598-017-07160-5 (PMC5537360; doi:10.1038/s41598-017-07160-5)
Supplement: Supplementary file 1 — Supplementary Information [file 41598_2017_7160_MOESM1_ESM.pdf]

Supplementary Information for:

Title: A 4D view on the evolution of metamorphic dehydration reactions

By: John Bedford, Florian Fusseis, Henri Leclère, John Wheeler, and Daniel Faulkner

This document includes:

Supplementary Figures 1 to 5

Supplementary Equations

Figure captions for Supplementary Movies 1 and 2

Supplementary Figure 1

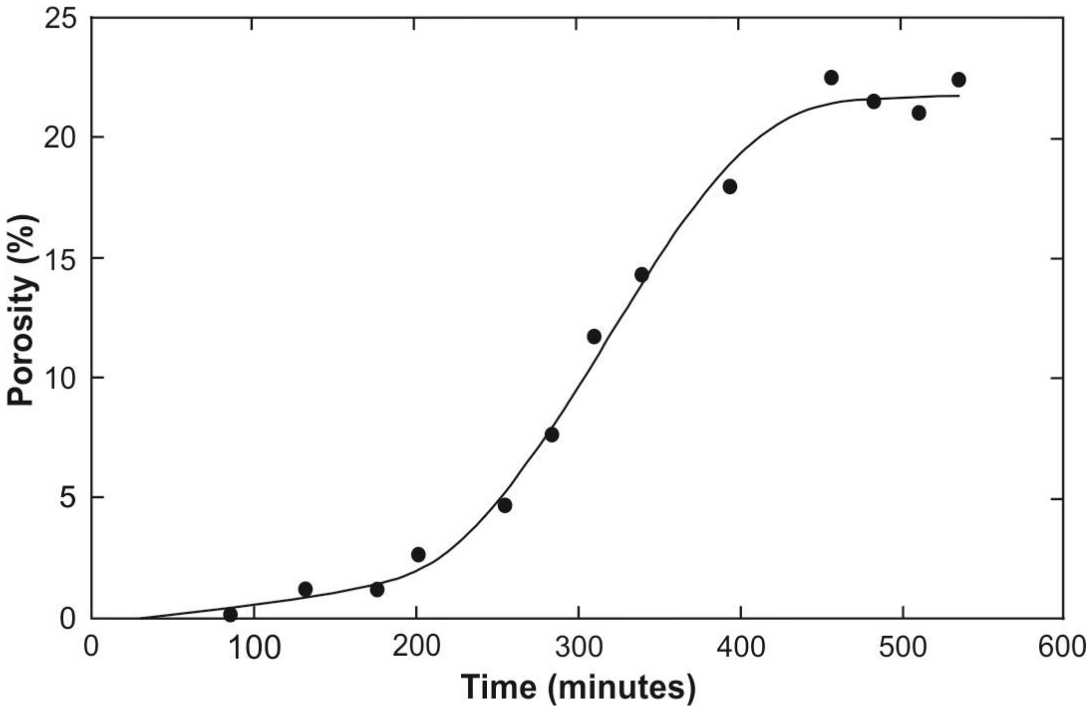

Supplementary Figure 1 | Porosity of the sample over time. Porosity increases non-linearly during the experiment until it settles around 22.5%.

Supplementary Figure 2

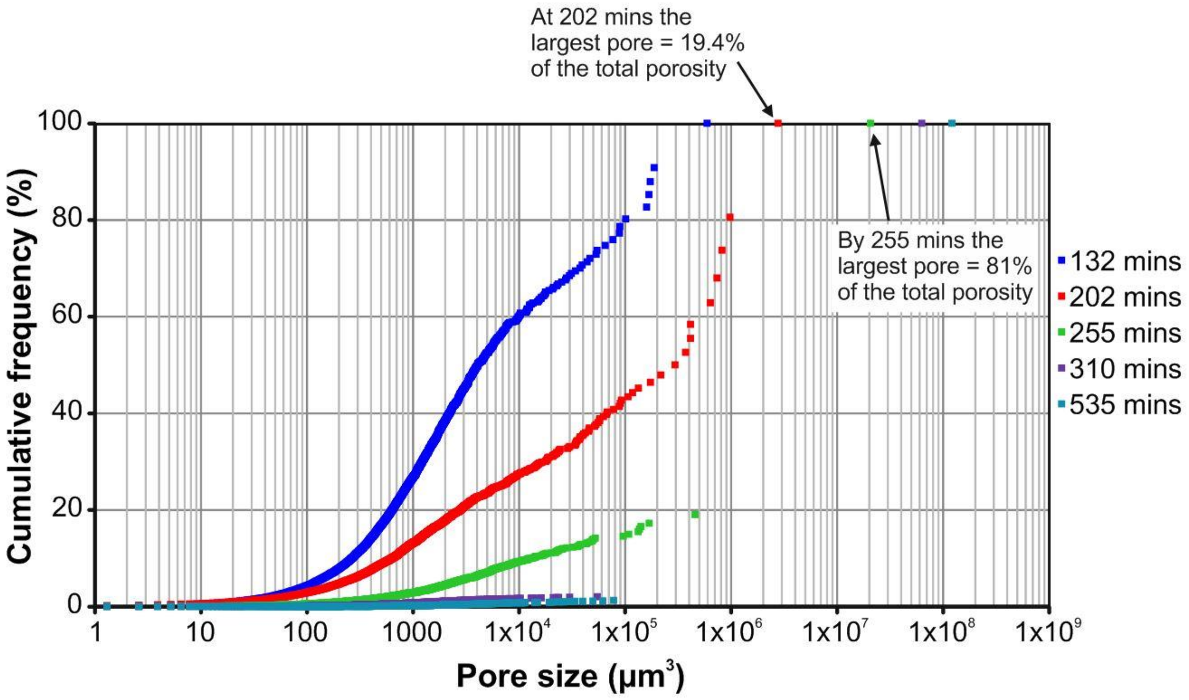

Supplementary Figure 2 | Cumulative pore size frequency distribution over time. After 255 minutes the distribution is dominated by a single cluster of interconnected pores which comprises more than 80% of the total porosity.

Supplementary Figure 3

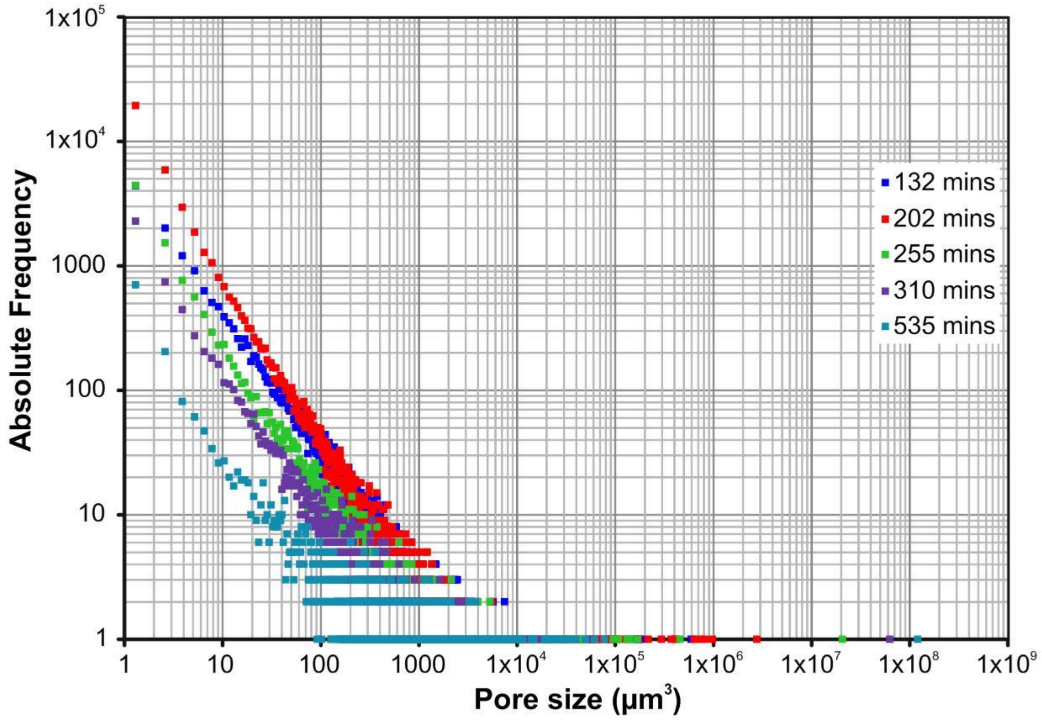

Supplementary Figure 3 | Absolute pore size frequency distribution over time. After 202 minutes the amount of small isolated pores drastically reduces as they become incorporated into the large sample-scale drainage architecture.

Supplementary Figure 4

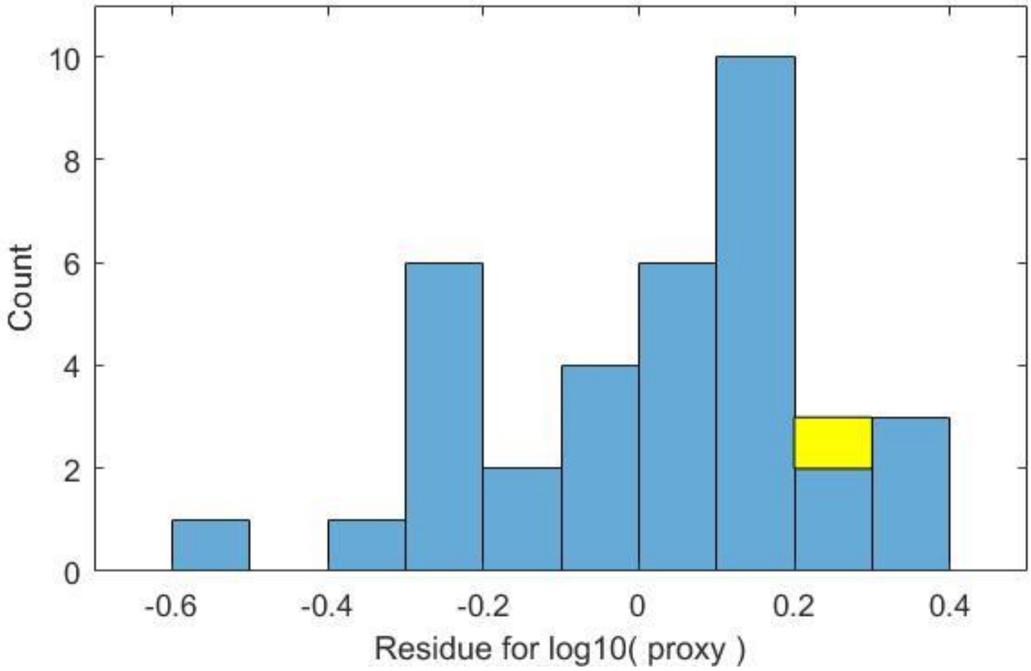

Supplementary Figure 4 | The scatter of residues of the measured minus calculated reaction proxy from the Llana-Funez et al., (2012) dataset. The data from this study is highlighted in yellow and is well within the scatter of residues from the Llana-Funez dataset. See supplementary equations for calculations and further information.

Supplementary Figure 5

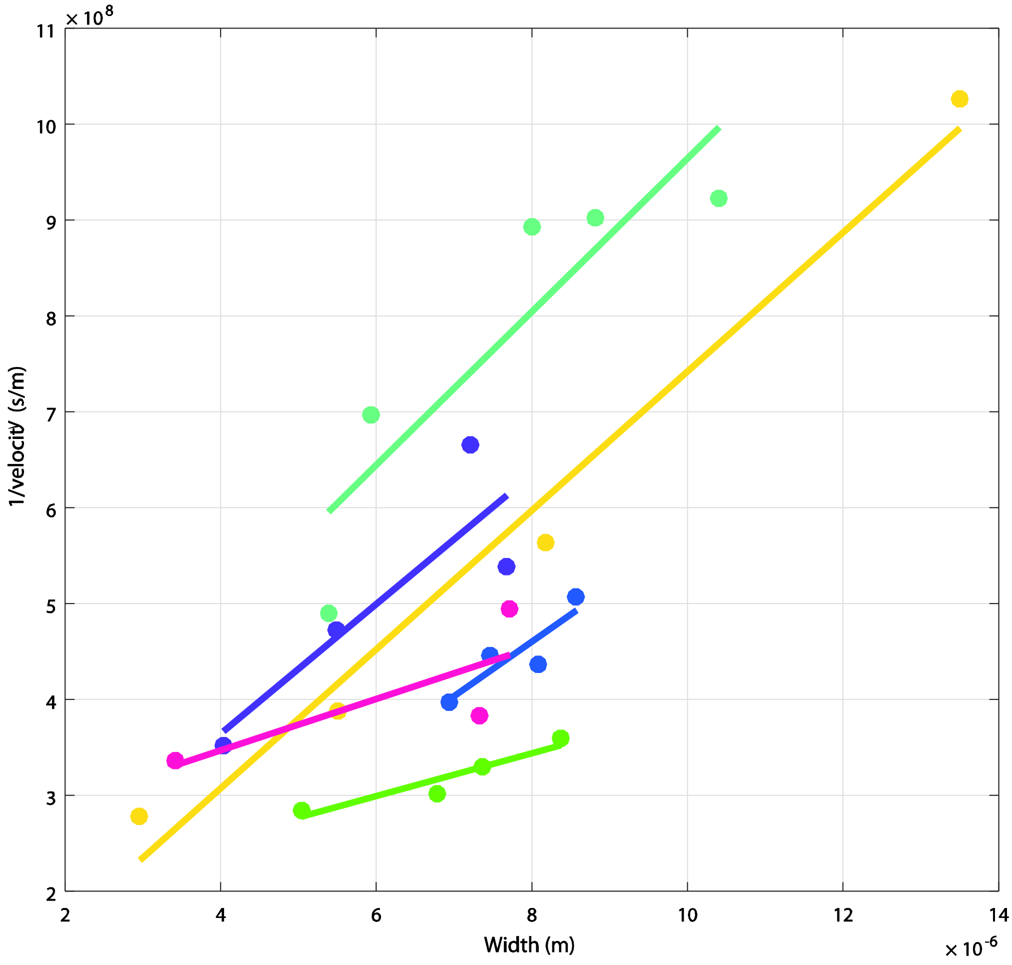

Supplementary Figure 5 | A plot of 1/velocity versus moat width for different grains. The slope of the best fit lines is used to derive the diffusion coefficient (D) in supplementary equation 2.

Supplementary Equations

1. ***Reaction rate is in agreement with a previously determined dependence on P and T***

Llana-Funez et al. (2012) took a dataset of reaction rates from 35 experiments on gypsum dehydration, using the volume of fluid expelled as a monitor of reaction rate. The maximum rate of fluid expulsion could be used as a measure of reaction rate but, realising that compaction also plays a role in fluid expulsion, they proposed that a reaction rate proxy is more useful:

Reaction rate proxy = (maximum expulsion rate)/(volume expelled at that time)

This idea can applied in the same way to porosity evolution. Because porosity development should scale with fluid expelled, for our new experiment we can calculate the same proxy using:

Reaction rate proxy = (maximum rate of porosity increase)/(porosity at that time)

Smoothing the data of Supplementary Figure 1 we find the maximum rate of porosity increase is 0.1267 %/min when porosity is 10.17 %. Then, converting to s^{-1},

log10(reaction rate proxy) = -3.68

Llana-Funez et al. made a best fit to their 35 reaction rate proxies, which span 2 order of magnitude:

$\log_{10}$(reaction rate proxy) = -16.9851 + 0.1142 T - 0.0127 P_f + 0.0019 P_c

So using our experimental parameters of Pc = 9 MPa, Pf = 4 MPa and T = 115 °C, we calculate

log10(reaction rate proxy) = -3.89

The difference of 0.21 is well within the scatter of residues (measured – calculated) in the Llana-Funez et al. dataset. Using their Supplementary Table 3, we display the residues in supplementary figure 4; the new result is shown in yellow and is not out of place in comparison to the overall scatter.

2. Determination of the diffusion coefficient

For figure 4 we required individual grain areas but it was not possible to segment bassanite from gypsum reliably: although the eye can distinguish these two minerals, there is too much overlap in grey scale. So, to analyze grain growth we selected individual grains which could be identified through the time series and measured the areas (A) and perimeters (p) of grains and moats in Fiji. We then calculated average growth and dissolution velocities by from $u = \frac{1}{p}\frac{dA}{dt}$. This approach was chosen because it guarantees the correct *average* growth velocity for a particular grain or moat. The net diffusion coefficient quoted in main text was calculated as follows. We assume that the growth rate is controlled by a combination of interface detachment from gypsum, attachment to bassanite and by diffusion leading to a simple quantitative model for rate[35]. We adapt eqns 9 and 10 from there but growth is 2D so there some convergence in chemical flux towards the bassanite. To allow for this we simplify grain and moat shapes to be cylinders. Then diffusive flux will scale with 1/r where r is radial distance from the center line of a bassanite grain. Concentration will be a linear function of ln(r) because its gradient gives the flux. Modifying and rearranging Lasaga (1986) eqns 9 and 10[(35)] gives:

$$\frac{1}{u} = \frac{\alpha \ln(\frac{1}{\alpha})}{(1-\alpha)V(C^g - C^b)D} w + \frac{1}{V(C^g - C^b)}\left(\frac{\alpha}{k_g} + \frac{1}{k_b}\right)$$ **Supp. eqn (1)**

Where:

u = bassanite growth speed

V = bassanite molar volume

w = moat width

C^g, C^b = concentrations of $CaSO_4$ in solution in equilibrium with gypsum or bassanite

k_g, k_b = rate constants for dissolution of gypsum and precipitation of bassanite, assuming rates are

linear in over- or undersaturation (m/s)

α^2 = (bassanite molar volume)/ (gypsum molar volume) = 0.71

So, D is derived from the slope s of a best fit line on a 1/u versus w graph:

$$D = \frac{\alpha \ln\left(\frac{1}{\alpha}\right)}{(1-\alpha)} \frac{1}{V(C^g - C^b)s}$$ **Supp. eqn (2)**

We applied this equation to 16 moats. For some moats the correlation coefficient was low so we excluded them; for others the y-axis intercept was negative which is not in accord with supplementary eqn (1) so we excluded these too. We were left with 6 moats with the data shown in supplementary figure 5. Each moat yields an estimate of D and the average is 1.23 $\times 10^{-10}$ m^2/s, the apparent diffusion coefficient of $CaSO_4$. Since the anion and cation have their own diffusion coefficients this is also the harmonic mean of the diffusion coefficients of Ca^{2+} and $SO4^{2-}$[36]. For comparison at 1 atm and 25 °C the apparent diffusion coefficient of $CaSO_4$ is 9.11 $\times 10^{-10}$ m^2/s[37]. We obtain a similar order of magnitude, but we expect the diffusion coefficients to be faster at higher T; the discrepancy may be due to a pressure effect and/or diffusion pathways which are longer than the moat width.

References

35 Lasaga, A. C. Metamorphic Reaction Rate Laws and Development of Isograds. Mineralogical Magazine **50**, 359-373 (1986).

36 Cussler, E. L. *Diffusion: Mass transfer in fluid systems*. (Cambridge University Press, 1984).

37 Yuan-Hui, L. & Gregory, S. Diffusion of ions in sea water and in deep-sea sediments. *Geochimica et Cosmochimica Acta* **38**, 703-714 (1974).

Figure captions for Supplementary Movies

Supplementary Movie 1 | A complete time series movie of the microtomographic reconstructions. The movie is of an individual 2D slice though the cylindrical sample. Sample diameter = 2 mm.

Supplementary Movie 2 | A 3D time series movie of the largest pore cluster. The analysed subvolume is a cube with a length of 975 µm. The pore cluster incorporates the majority of individual pores into sample-scale drainage architecture after 255 minutes.
